# Developing and pilot testing an oral health screening tool for diabetes care providers

**DOI:** 10.1186/s12875-022-01798-5

**Published:** 2022-08-11

**Authors:** Ajesh George, Prakash Poudel, Ariana Kong, Amy Villarosa, Hanny Calache, Amit Arora, Rhonda Griffiths, Vincent W. Wong, Mark Gussy, Rachel E. Martin, Phyllis Lau

**Affiliations:** 1 Australian Centre for Integration of Oral Health (ACIOH), School of Nursing & Midwifery, Western Sydney University, Liverpool, NSW 1871 Australia; 2 Ingham Institute for Applied Medical Research, Liverpool, NSW 1871 Australia; 3 School of Dentistry, Faculty of Medicine and Health, The University of Sydney, Surry Hills, NSW 2010 Australia; 4 South Western Sydney Local Health District, Liverpool, NSW 1871 Australia; 5 La Trobe Rural Health School, La Trobe University, Bendigo, VIC 3552 Australia; 6 Translational Health Research Institute, Western Sydney University, Campbelltown, NSW 2560 Australia; 7 School of Health and Social Development, Faculty of Health, Institute for Health Transformation, Deakin University, Burwood, VIC 3125 Australia; 8 Melbourne Dental School, The University of Melbourne, Carlton, VIC 3053 Australia; 9 School of Health Sciences, Western Sydney University, Campbelltown, NSW 2560 Australia; 10 Health Equity Laboratory, Campbelltown, NSW 2560 Australia; 11 Discipline of Child and Adolescent Health, Sydney Medical School, The University of Sydney, Camperdown, NSW 2050 Australia; 12 Sydney Dental Hospital/Sydney Local Health District, Surry Hills, NSW 2010 Australia; 13 School of Nursing & Midwifery, Western Sydney University, Penrith, NSW 2751 Australia; 14 South-Western Sydney Clinical School, University of New South Wales, Liverpool, NSW 2170 Australia; 15 Lincoln International Institute for Rural Health, University of Lincoln, Lincoln, United Kingdom; 16 School of Population and Global Health, The University of Melbourne, Melbourne, VIC 3010 Australia; 17 School of Medicine, Western Sydney University, Campbelltown, NSW 2560 Australia; 18 Department of General Practice, The University of Melbourne, Melbourne, VIC 3010 Australia

**Keywords:** Diabetes, Oral health, Screening, Health personnel, Periodontal disease

## Abstract

**Background:**

People with poorly managed diabetes are at greater risk of periodontal disease. Periodontal disease that is not effectively managed can affect glycaemic levels. Diabetes care providers, including general practitioners and diabetes educators, are encouraged to promote oral health of their clients. However, valid and reliable oral health screening tools that assess the risk of poor oral health, that are easy to administer among non-dental professionals, currently do not exist. Existing screening tools are difficult to incorporate into routine diabetes consultations due to their length. Thus, this study aimed to develop and pilot a short oral health screening tool that would identify risk of existing oral diseases and encourage appropriate referrals to the dental service.

**Methods:**

A three-item screening tool was developed after a comprehensive review of the literature and consensus from an expert panel. The tool was then piloted as part of a larger cross-sectional survey of 260 adults with diabetes who were accessing public diabetes clinics at two locations in Sydney, Australia. As part of the survey, participants completed the three-item screening tool and a 14-item validated tool, the Oral Health Impact Profile (OHIP-14), which has been used previously in the preliminary validation of screening tools. Sensitivity and specificity analyses were then undertaken comparing the results of the two tools.

**Results:**

A statistically significant correlation was found between the shorter screening tool and the OHIP-14 (rho = 0.453, *p* < 0.001), indicating adequate validity. The three-item tool had high sensitivity (90.5%, 95% CI 84.9%, 94.7%), with a specificity of 46.3% (95% CI 37.7%, 55.2%). The negative predictive value was 81.4% (95% CI 71.3, 89.3). No single item performed as well regarding sensitivity and negative predictive value when compared to the three items collectively.

**Conclusions:**

The three-item screening tool developed was found to be valid and sensitive in identifying risk of poor oral health, requiring oral health referrals, among people with diabetes in this pilot. This is a simple, accessible tool that diabetes care providers could incorporate into their routine consultations. Further validation against comprehensive dental assessments is needed to reassess the tool’s specificity and sensitivity in diverse settings.

## Background

Diabetes mellitus is a metabolic disorder characterised by elevated blood glucose levels (hyperglycaemia) due to insulin deficiency or insulin resistance [1]. Diabetes is considered a significant global health issue [2, 3] and is referred to as the world’s largest non-communicable epidemic [4, 5]. The global prevalence of diabetes was 8.5% in 2014 [3], and it is predicted that approximately 700 million people will be living with diabetes by 2045 [6]. In Australia, around 1.2 million individuals are diagnosed with diabetes, with more than 10,000 new cases identified every year [7]. The economic burden of diabetes to the Australian economy is estimated to be over $6 billion annually [8].

Chronic and poorly managed diabetes affects an individual’s cardiovascular, renal, optical, neurological and oral health [9]. The relationship between diabetes and oral health is significant [10] with a bidirectional link between hyperglycaemia and periodontitis (advanced periodontal disease) [1]. Specifically, periodontitis can adversely affect diabetes management as periodontitis is linked to an increase in glycated haemoglobin (HbA1C) [11]. Individuals with poorly managed diabetes are up to three times more likely to develop periodontitis than individuals without diabetes [5] and are also at an increased risk of dry mouth, tooth decay, tooth loss, oral thrush and taste impairment [12]. Systematic reviews and meta-analyses suggest that a significant reduction in HbA1C ranging from 0.3–0.7% [13–17] is observed among individuals with diabetes who have received periodontal treatment.

Despite the literature providing evidence detailing the importance of oral health in diabetes management, studies suggest that there is a gap in oral health knowledge, attitudes and practices among people with diabetes. One systematic review found that individuals were generally not aware of the relationship between diabetes and oral health [18]. Furthermore, attitudes towards oral health, as well as compliance with the recommended oral hygiene practices and dental visits were poor [18]. A review of studies worldwide found that just over half of the people with diabetes had visited a dentist in the last 12 months with dental costs cited as the main underlying barrier to lower dental visits [18]. Impaired oral health is already known to affect quality of life [19]. Individuals with diabetes who also experience mouth pain, xerostomia, halitosis and periodontitis, report lower oral health-related quality of life (OHQoL) and overall health-related quality of life compared to people without diabetes [20]. Nevertheless, there is evidence to suggest that people with diabetes are receptive to advice about oral health from diabetes care providers such as diabetes educators and general practitioners (GPs) [18, 21]. Receiving such advice has been shown to significantly improve the oral health behaviours like brushing frequency and dental visits among patients with diabetes [22, 23].

The role of diabetes care providers, particularly diabetes educators, is to provide guidance and education on the management of diabetes [24]. Current clinical practice guidelines for the prevention and management of diabetes also recommend the integration of an oral health review and referral by diabetes care providers [25–27]. Diabetes care providers are therefore ideally placed to address the gaps in knowledge and behaviours with respect to oral health among clients. However, a review of current literature has shown very few Diabetes care providers discuss oral health and there are significant barriers for them to undertake this role which include lack of training in this area, time constraints, lack of appropriate oral health referral pathways as well as limited oral health promotional resources and screening tools [2, 24].

Over the years various strategies have been implemented in Australia to support diabetes care providers to promote oral health. These have included professional development oral health training programs, tailored evidenced based resources for people with diabetes and government schemes to improve access to oral health services [28–30]. To further assist diabetes care providers in screening and referring clients to oral healthcare professionals, recent needs assessment studies (involving diabetes educators and GPs) in Australia [2, 24] revealed the need for a short, non-invasive oral health screening tool. Diabetes care providers felt that including such a tool as part of their normal assessment protocol would provide a trigger for them to discuss oral health with their clients [24] and provide prompt screening and dental referrals. Shorter screening tools have been developed for non-dental professionals in other settings. A short two-item oral health screening tool was developed by George et al. [31] for midwives to screen and refer pregnant women to the dentist. This validated tool was found to have high sensitivity (up to 94%) [32], and was easily administered by midwives [31]. Nijland et al. [33] also developed an 8-item screening tool for medical professionals to screen for periodontitis, although it had a comparatively lower sensitivity (49%). Among people with diabetes, however, there are currently no short, screening tools available to screen for risk of poor oral health. Validated tools such as the 21-item Diabetes Oral Health Assessment Tool (DiOHAT) [34] and the 17-item modified DiOHAT [35] have been identified as difficult to incorporate into practice due to their length and time constraints. The aim of this study, therefore, was to develop and pilot a short oral health screening tool for diabetes care providers.

## Methods

### Development of the oral health screening tool

**Fig. 1:**
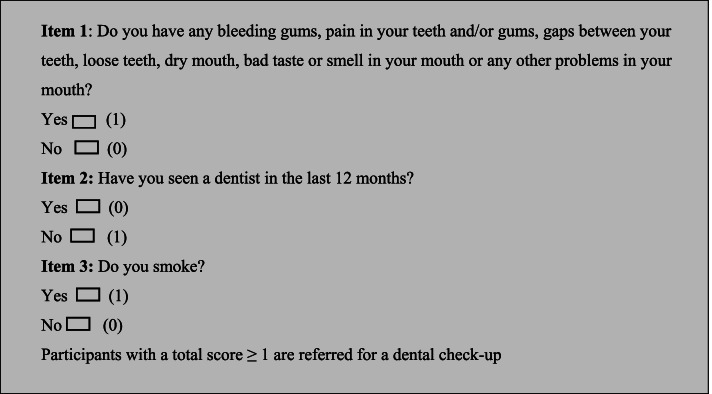
Oral health screening tool

A comprehensive review of the literature was undertaken to identify potential items for inclusion in the screening tool. The review particularly focussed on existing oral health screening tools for diabetes as well as shorter tools used for other disorders. The results generated three potential items of which two were tested and validated in other population groups. These items were then reviewed by an expert panel consisting of academics and clinicians from relevant fields (dentistry, diabetes, general practice and public health) as well as among people with diabetes. The items were then revised to reflect the needs of people with diabetes and the Australian context (Fig. 1). The first item related to commonly reported oral health problems encountered with diabetes and those caused by related dental diseases, such as periodontitis. The second item focussed on the frequency of dental visits in the last 12 months. The last item related to smoking, and was included as it is a known risk factor for both Type 2 diabetes and periodontitis [36, 37]. The items were scored 0 and 1 and a total of score of ≥ 1 (having a dental problem or not seen a dentist in the last 12 months or currently smoking) indicated the person with diabetes was at risk of poor oral health and required a referral to an oral health professional (Fig. 1).

### Testing of the screening tool

The tool was tested as part of a cross-sectional survey that assessed the self-reported oral health status, knowledge and behaviours of people living with diabetes. Additional information about this survey has been published elsewhere by Poudel et al. [38]. The data pertaining to the oral health screening tool was used for the analysis in this study.

### Sample and setting

Adults who have been diagnosed with diabetes (type 1 or type 2) and were accessing one of four public diabetes clinics in Sydney, Australia, were eligible to participate in the study. For participants who were not fluent in English, accredited interpreters were used where possible, or family members were asked to assist the participant to complete the questionnaire. People who were not comfortable communicating in English and did not have the assistance of an interpreter or family member were excluded from the study.

Participants were recruited from one hospital clinic in northern Sydney as well as three hospital clinics in South Western Sydney. These two regions were selected as they capture a diverse range of socioeconomic backgrounds. The local government area (LGA) covering South Western Sydney includes populations that are more disadvantaged, and reports one of the highest rates of diabetes across Sydney [39]. The LGA for northern Sydney, however, represents one of the most advantaged populations in Sydney [40].

### Recruitment and procedure

Information about the study was advertised through flyers posted in waiting rooms of the diabetes clinics. An experienced investigator (PP) attended each diabetes clinic to provide potential participants with additional information about the study, including a summary of the study aims and protocol. The presence of the investigator on site provided an opportunity for potential participants to ask any questions before providing written consent to participate. Participants completed the self-administered questionnaire prior to their appointment while in the waiting room. Oral health promotion information and dental products (toothbrush and toothpaste) were provided to all invited participants, irrespective of whether they took part in the study.

### Data collection

Data collection of the surveys occurred between March 2019 and January 2020. Each participant that completed the questionnaire also completed both the three-item diabetes and oral health screening tool and the Oral Health Impact Profile-14 (OHIP-14) [41]. Participants typically took about 10 to 15 min to complete the questionnaire. The OHIP-14 tool was developed in 1994 and is a valid and reliable instrument that provides a subjective measure of oral health and its impact on quality of life [41]. Numerous studies have demonstrated a significant association between the OHIP-14 tool and poor oral health [23, 42] in particular periodontal disease [43]. Further, measuring oral health quality of life using the tool can inform the dental care needs of patients and help health care providers in treatment planning [44, 45]. The OHIP-14 tool has been used in previous studies for preliminary validation of oral health screening tools for other populations like HIV patients and pregnant women [32, 46]. It has also been used in other studies to assess the oral health quality of life, including among people with diabetes [47, 48]. The OHIP-14 tool asks respondents 14 questions across 7 domains (functional limitation, physical pain, psychological discomfort, physical disability, psychological disability, social disability and handicap) that are measured using a Likert rating scale from 0 (“never”) to 4 (“very often”). The sum of all items were added for a score out of a total possible of 56, where higher scores reflected poorer self-reported oral health [41]. The responses for the oral health screening tool (Fig. 1) used three items with dichotomous variables (‘Yes’ and ‘No’) for responses, with responses corresponding with a score of either one or zero. The sum of each item was added, with scores ranging from zero to three. As indicated in the tool (Fig. 1), individuals who scored one or greater were indicated for a dental referral.

Demographic information about participants were also collected. These questions asked about the person’s gender, cultural and linguistic background, type of diabetes, lifestyle behaviours, presence of other chronic diseases, employment, education, marital status, annual household income, private health insurance and whether they had a government-issued concession card.

This study was approved by the South Western Sydney Local Health District Research and Ethics Committee (LNR/18/LPOOL/510), Northern Sydney Local Health District Research Office (RESP/19/017) and the Human Research Ethics Committee of Western Sydney University (RH13145). The study was also conducted in accordance with the Australian National Health & Medical Research Council’s national statement on ethical conduct in human research, which follows the principles outlined in the Declaration of Helsinki.

### Data analysis

Data were manually entered and analysed using SPSS (Version 27) Demographic data were analysed for descriptive statistics. As both OHIP-14 and the Oral Health screening tool scores were not normally distributed, these scores were correlated using Spearman’s rank-order correlation coefficient (rho). Following this, both variables were dichotomised for further analysis. With no existing cut-offs developed for OHIP-14, this variable was dichotomised at the median according to the method outlined by Locker et al. [49]. The oral health screening tool was dichotomised as 0 = 0 (no risk), and any score greater than or equal to 1 = 1 (at risk). As the purpose of this screening tool was to identify individuals at risk of poor oral health, high sensitivity was crucial to ensure as many at-risk individuals as possible were identified. Thus, the at-risk score of 1 or greater was chosen to optimise sensitivity over specificity. Sensitivity analyses were conducted to evaluate the dichotomised oral health screening tool against the dichotomised OHIP-14 scores, whereby sensitivity, specificity, positive predictive value and negative predictive value were calculated. Additional sensitivity analysis was undertaken to test each individual oral health screening tool items against the OHIP-14. For all analyses, alpha was set at 0.05.

## Results

A total of 260 patients completed the survey. The average age of participants was 61.7 ± 13.8 years, and over half (*n* = 139, 53.5%) of participants were male. More than two thirds (*n* = 177, 68.1%) of participants were born overseas, and 57.7% (*n* = 150) spoke English at home. Most participants (*n* = 224, 86.1%) reported educational attainment of at least secondary school. More than half of participants (*n* = 143, 55.0%) had a combined household income of less $40,000, and over two-thirds (*n* = 177, 68.1%) did not have private health insurance, with over half (*n* = 151, 58.0%) eligible for public dental services via health care card or being a defence force veteran. The majority of participants (*n* = 226, 86.9%) reported having type 2 diabetes, with the mean duration since their diagnoses being 15.3 (SD 11.66) years. Within the oral health screening tool, 53.1% (*n* = 138) of responses indicated having dental problems in Item 1, 37.3% (*n* = 97) of responses in Item 2 indicated they had not visited a dentist in the last 12 months, and 14.2% of responses (*n* = 37) indicated they were smokers in Item 3. A total of 190 participants (73.1%) scored one or higher on this tool, indicating that they would require a dental referral. According to the OHIP-14, 52.7% (*n* = 137) of participants scored 21 or higher, and thus were indicated for a dental visit.

Spearman’s rank-order correlations revealed a statistically significant correlation between the oral health screening tool and the OHIP-14 (rho = 0.453, *p* < 0.001), indicating validity of the oral health screening tool. Furthermore, the results of the sensitivity analyses indicated that the 3-item oral health screening tool had high sensitivity (90.5%, 95% CI 84.9%, 94.7%), with a specificity of 46.3% (95% CI 37.7%, 55.2%). The negative predictive value was 81.4% (95% CI 71.3, 89.3). For individual items, Item 1 had the highest sensitivity 79.6% (95% CI 72.3%, 85.7%) and Item 3 had the lowest (18.2%, (95% CI 12.4%, 25.3%), but Item 3 had the highest specificity at 90.2% (95% CI 84.2%, 94.7%) and Item 2 having the lowest (66.7%, 95%CI 58.1%, 74.6%). No single item performed as well regarding sensitivity and negative predictive value when compared to the three items collectively (Table 1).

**Table 1:** Comparison of sensitivity, specificity, positive predictive value and negative predictive value for the screening tool

	3-Item oral health screening tool	Item 1 (Dental problems)	Item 2 (Dental attendance)	Item 3 (Smoking)
	% (95% CI)	% (95% CI)	% (95% CI)	% (95% CI)
Sensitivity	90.5 (84.9—94.7)	79.6 (72.3—85.7)	40.9 (32.9—49.2)	18.2 (12.4—25.3)
Specificity	46.3 (37.7—55.2)	76.4 (68.4—83.3)	66.7 (58.1—74.6)	90.2 (84.2—94.7)
Positive predictive value	65.3 (58.3—71.8)	79 (71.7—85.2)	57.7 (47.8—67.3)	92.3 (51.7—81.1)
Negative predictive value	81.4 (71.3—89.3)	77 (69.1—83.9)	50.3 (42.7—57.9)	49.8 (43.2—56.3)

## Discussion

This study aimed to develop and pilot a short oral health screening tool for diabetes care providers to utilise in routine clinical practice. A key aspect of a screening tool is its validity. In this study the sensitivity of each item varied from 18.2% to 79.6%, but collectively the three items showed marked improvement in sensitivity (90.5%). The three items also had a significant correlation with the OHIP-14 indicating adequate validity. This is particularly important as poor oral health like periodontal disease has been found to be significant correlated with higher OHIP-14 scores [50]. These are very positive findings that could be attributed to the way the items were developed. The first item explored the most common dental problems linked with poor oral health, and more than half of the respondents reported (53%) these concerns (dry mouth, gaps between teeth, pain in teeth and/or gums and loose teeth) in the related study surveying people with diabetes in Australia [38]. The second item related to the frequency of dental visits in the last 12 months as the evidence shows that infrequent dental attendance is associated with poor oral health [51]. Smoking was included as the last item as it is a risk factor for both diabetes and periodontal disease [36, 37]. A recent systematic review and meta-analysis found that smoking increases the risk of periodontal disease by 80% [52]. The combination of the three items also improved the negative predictive value (NPV) (81.4%) suggesting the tool is reliable in excluding dental referrals for people with diabetes deemed not at risk of poor oral health. This is an important factor as a high NPV is desirable in conditions where early intervention is advisable and effective [53]. It is well established that people with uncontrolled diabetes are at greater risk of periodontitis, which can deteriorate very rapidly [5, 10]. Thus, early detection of periodontitis and subsequent dental treatment can improve periodontal health as well as glycaemic control. Studies show periodontal treatment can decrease HbA1c levels by 0.3–0.7% which is similar to adding a second oral anti-diabetic medication to metformin [25, 54]. Seeing a dentist earlier can also reduce the cost of associated treatment [55].

One issue that is evident in the findings is the lower specificity (46.3%) and positive predictive value (PPV) (65.3%) when combining the three items. This is not surprising as the sensitivity of any diagnostic tool is influenced by the prevalence of the disease [56]. A meta-analysis 23 studies of analysing diagnostic accuracy found that specificity significantly reduced with higher prevalence [56]. These findings are relevant to the current study as there is a high prevalence of dental problems among people with diabetes ranging from 53–86% depending on the demographic profile [38, 57, 58]. Low specificity and NPV is an issue for screening tools if there are cost implications or risk of harm and discomfort to the patient from treatment [53]. However, in the case of oral health screening for patients with diabetes this may not be an issue. The current consensus worldwide is that all people with diabetes should have regular dental check-ups as part of diabetes self-management [25, 27, 59]. Thus, people with diabetes, who may not have any oral disease, but are incorrectly referred to an oral health professional through this screening process will still have a thorough oral health examination to assess their oral health status. These benefits could justify a tool having moderate PPV [53] particularly if there is potential flow-on health benefits such as improved glycaemic control [54] and quality of life [60] through preventive and timely dental examinations. Having a “dental home” is an important element of health care for people with diabetes, as the oral health professional is able to make a more specific risk assessment from the dental examination to help tailor preventive care and inform the timing of review examinations for the patient [61].

Lastly, in addition to validity it is important to consider the acceptability of the screening tool for both the diabetes care providers and their patients. Previous research in Australia has shown that diabetes educators and GPs prefer short screening tools due to their time constraints [2, 24]. Further a recent doctoral study surveyed the views of 260 patients with diabetes in Sydney and found that more than 88% were receptive to diabetes care providers asking questions about their oral health and initiating dental referrals [62]. These initial studies suggest that the tool could be acceptable for all stakeholders, but more research is needed to confirm that it can be implemented into practice and is easy to use in diabetes care settings without evoking any discomfort from patients.

### Limitations

This study is limited by the non-random sampling method which could have led to a biased selection of participants who were well motivated in oral health care. The sample only included people with type 1 and type 2 diabetes, not people with gestational diabetes. Further, the study was undertaken across metropolitan Sydney, Australia and thus the findings may differ in other populations of people with diabetes from regional/rural areas as well as other countries. However, all attempts were made to recruit diverse participants from across the socio-economic spectrum in Sydney including those from culturally and linguistically diverse backgrounds and the resulting sample was fairly representative of the population data from the Australian National Diabetes Audit [38]. Another limitation is that only one aspect of the psychometric assessment of the tool was assessed and other aspects like construct validity was not undertaken. Lastly, the OHIP-14 may not be the ideal standard to validate the tool and as this study did not utilise clinical examinations for validation, correlations with actual oral health status remain unknown. Despite this, the OHIP-14 is likely a sound standard due to its known correlation with poor oral health and dental care needs.

## Conclusion

Diabetes care providers are recommended to undertake oral health risk assessments of their clients but currently there are no simple screening tools that can assist them in this role. This study has identified a simple 3-item screening tool with high sensitivity and NPV that can identify risks of poor oral health which requires dental referrals in patients with diabetes, without adding extra burden on the consultation time for the clinician. This tool has the potential to be easily incorporated into diabetes care practice but further validation is needed using against comprehensive dental assessments of screened clients as the gold standard.

## Data Availability

Data are available from the corresponding author upon request.

## References

[1] Lamster IB, Lalla E, Borgnakke WS, Taylor GW (2008). The relationship between oral health and diabetes mellitus. J Am Dent Assoc.

[2] Poudel P, Griffiths R, Wong VW, Arora A, Flack JR, Khoo CL, George A (2020). Perceptions and practices of general practitioners on providing oral health care to people with diabetes - a qualitative study. BMC Fam Pract.

[3] World Health Organization: Diabetes - key facts. WHO. 2021. https://www.who.int/news-room/fact-sheets/detail/diabetes. Accessed 5 Nov 2021.

[4] Shaw JE, Sicree RA, Zimmet PZ (2010). Global estimates of the prevalence of diabetes for 2010 and 2030. Diabetes Res Clin Pract.

[5] Chapple IL, Genco R, Working Group 2 of the Joint EFP/AAP Workshop (2013). Diabetes and periodontal diseases: consensus report of the joint EFP/AAP workshop on periodontitis and systemic diseases. J Periodontol.

[6] International Federaton of Diabetes: Diabetes facts and figures IDF. 2020. https://www.idf.org/aboutdiabetes/what-is-diabetes/facts-figures.html. Accessed 8 Aug 2020.

[7] National Diabetes Services Scheme: Data snapshots. NDSS. 2015. https://static.diabetesaustralia.com.au/s/fileassets/diabetes-australia/7f605325-256c-4736-adc1-75a3b5c3fa20.pdf. Accessed 8 Aug 2020.

[8] Baker IDI Heart and Diabetes Institute. Diabetes: the silent pandemic and its impact on Australia. Diabetes Australia. 2012. https://www.diabetesaustralia.com.au/wp-content/uploads/Diabetes-the-silent-pandemic-and-its-impact-on-Australia.pdf. Accessed 11 Mar 2022.

[9] World Health Organization. Global report on diabetes. Geneva: WHO. 2016. https://www.who.int/publications/i/item/9789241565257. Accessed 5 Nov 2021.

[10] Taylor GW, Borgnakke W (2008). Periodontal disease: associations with diabetes, glycemic control and complications. Oral Dis.

[11] Preshaw PM, Bissett SM (2019). Periodontitis and diabetes. Br Dent J.

[12] Leite RS, Marlow NM, Fernandes JK, Hermayer K (2013). Oral health and type 2 diabetes. Am J Med Sci.

[13] Simpson  TC, Needleman  I, Wild  SH,  Moles  DR, Mills  EJ (2010). Treatment of periodontal disease for glycaemic control in people with diabetes. Cochrane Database Syst Rev.

[14] Darré L, Vergnes J-N, Gourdy P, Sixou M (2008). Efficacy of periodontal treatment on glycaemic control in diabetic patients: a meta-analysis of interventional studies. Diabetes Metab.

[15] Borgnakke WS, Ylöstalo PV, Taylor GW, Genco RJ (2013). Effect of periodontal disease on diabetes: systematic review of epidemiologic observational evidence. J Periodontol.

[16] Teeuw WJ, Gerdes VE, Loos BG (2010). Effect of periodontal treatment on glycemic control of diabetic patients: a systematic review and meta-analysis. Diabetes Care.

[17] Liew AKC, Punnanithinont N, Lee YC, Yang J (2013). Effect of non-surgical periodontal treatment on HbA1c: a meta-analysis of randomized controlled trials. Aust Dent J.

[18] Poudel P, Griffiths R, Wong VW, Arora A, Flack JR, Khoo CL, George A (2018). Oral health knowledge, attitudes and care practices of people with diabetes: a systematic review. BMC Public Health.

[19] Baiju RM, Peter E, Varghese NO, Sivaram R. Oral health and quality of life: current concepts. J Clin Diagn Res. 2017;11(6):ZE21-ZE6. 10.7860/JCDR/2017/25866.10110 PMC5535498 28764312

[20] Verhulst MJ, Teeuw WJ, Gerdes VE, Loos BG (2019). Self-reported oral health and quality of life in patients with type 2 diabetes mellitus in primary care: a multi-center cross-sectional study. Diabetes Metab Syndr Obes.

[21] Karikoski A, Ilanne-Parikka P, Murtomaa H (2002). Oral self-care among adults with diabetes in Finland. Community Dent Oral Epidemiol.

[22] Mirza KM, Khan AA, Ali MM, Chaudhry S (2007). Oral health knowledge, attitude, and practices and sources of information for diabetic patients in Lahore. Pakistan Diabetes Care.

[23] Yuen HK, Wolf BJ, Bandyopadhyay D, Magruder KM, Salinas CF, London SD (2009). Oral health knowledge and behavior among adults with diabetes. Diabetes Res Clin Pract.

[24] Poudel P, Griffiths R, Wong VW, Arora A, Flack JR, Khoo CL, George A (2018). Perceptions and practices of diabetes educators in providing oal health care: a qualitative study. Diabetes Educ.

[25] Sanz M, Ceriello A, Buysschaert M, Chapple I, Demmer RT, Graziani F, Herrera D, Jepsen S, Lione L, Madianos P (2018). Scientific evidence on the links between periodontal diseases and diabetes: consensus report and guidelines of the joint workshop on periodontal diseases and diabetes by the International Diabetes Federation and the European Federation of Periodontology. Diabetes Res Clin Pract.

[26] Royal Australian College of General Practitioners. General practice management of type 2 diabetes: 2016–18. East Melbourne, Victoria: RACGP. 2016. https://www.racgp.org.au/FSDEDEV/media/documents/Clinical%20Resources/Guidelines/Diabetes/General-practice-management-of-type-2-diabetes_1.pdf. Accessed 5 Nov 2021.

[27] IDF Clinical Guidelines Task Force. Guideline: oral health for people with diabetes. Internet. Brussels, Belgium: International Diabetes Federation. 2009. https://www.idf.org/e-library/guidelines/83-oral-health-for-people-with-diabetes. Accessed 5 Nov 2021.

[28] Dental Health Services Victoria: Diabetes educators. Carlton, Victoria: DHSV. https://www.dhsv.org.au/oral-health-advice/Professionals/diabetes-educators?SQ_DESIGN_NAME=print. Accessed 4 Apr 2022.

[29] Centre for Oral Health Strategy: Diabetes and oral health. Sydney, NSW: NSW Health. 2021. https://www.health.nsw.gov.au/oralhealth/prevention/Pages/diabetes-oral-health.aspx. Accessed 4 Apr 2022.

[30] Department of Health and Ageing: Information for patients: dental services under Medicare for people with chronic and complex conditions. Australia: Australian Government. 2010. https://www.spinal.com.au/wp-content/uploads/2011/02/Attachment-1.-Medicare.pdf. Accessed 4 Apr 2022.

[31] George A, Ajwani S, Johnson M, Dahlen H, Blinkhorn A, Bhole S, Ellis S, Zheng C, Dawes W (2015). Developing and testing of an oral health screening tool for midwives to assess pregnant woman. Health Care Women Int.

[32] George A, Dahlen HG, Blinkhorn A, Ajwani S, Bhole S, Ellis S, Yeo A, Elcombe E, Sadozai A, Johnson M (2016). Measuring oral health during pregnancy: sensitivity and specificity of a maternal oral screening (MOS) tool. BMC Pregnancy Childbirth.

[33] Nijland N, Overtoom F, Gerdes VEA, Verhulst MJL, Su N, Loos BG (2021). External validation of a rapid, non-invasive tool for periodontitis screening in a medical care setting. Clin Oral Investig.

[34] Kuwamura Y, Sumikawa M, Sakamoto E, Kishida S (2018). The utilization of the diabetes oral health assessment tool© for nurses by diabetes nurse specialists. J Nurs Investig.

[35] Kuwamura Y, Yoshida S, Kurahashi K, Sumikawa M, Sakamoto E, Aihara K-i, Yumoto H, Kuroda A, Endo I, Yasui T (2020). Modified diabetes oral health assessment tool (M‐DiOHATⓒ) for nurses and their association with efficacy beliefs and outcome expectancies in patients with diabetes. J Nurs Investig.

[36] Leite FRM, Nascimento GG, Scheutz F, López R (2018). Effect of smoking on periodontitis: a systematic review and meta-regression. Am J Prev Med.

[37] Pan A, Wang Y, Talaei M, Hu FB, Wu T (2015). Relation of active, passive, and quitting smoking with incident type 2 diabetes: a systematic review and meta-analysis. Lancet Diabetes Endocrinol.

[38] Poudel P, Griffiths R, Arora A, Wong VW, Flack JR, Barker G, George A (2021). Oral health status, knowledge, and behaviours of people with diabetes in Sydney, Australia. Int J Environ Res Public Health.

[39] HealthStats NSW: Diabetes prevalence in adults. NSW: NSW Government. 2020. http://www.healthstats.doh.health.nsw.gov.au/Indicator/dia_prev_age/dia_prev_lhn. Accessed 11 Mar 2022.

[40] Sydney North Primary Health Network: North Sydney local government area health profile. Sydney: SNPHN. 2016. https://sydneynorthhealthnetwork.org.au/wp-content/uploads/2016/12/SNPHN-LGA-fact-sheet-North-Sydney-NEW-151216.pdf. Accessed 11 Mar 2022.

[41] Slade GD, Spencer AJ (1994). Development and evaluation of the oral health impact profile. Community Dent Health.

[42] Warsi I, Younus A, Rasheed A, Ahmed J, Mahida H, Hashmi R, Qureshi A (2018). Oral health-related quality of life in patients with upper gastrointestinal and hepatic disorders in Pakistan: validation of the Oral Health Impact Profile-14 in the Urdu language. BDJ Open.

[43] Ng SK, Leung WK (2006). Oral health-related quality of life and periodontal status. Community Dent Oral Epidemiol.

[44] Mary AV, Mahendra J, John J, Moses J, Ebenezar AVR, Kesavan R (2017). Assessing quality of life using the Oral Health Impact Profile (OHIP-14) in subjects with and without orthodontic treatment need in Chennai, Tamil Nadu, India. J Clin Diagnos Res.

[45] Sheng X, Xiao X, Song X, Qiao L, Zhang X, Zhong H (2018). Correlation between oral health and quality of life among the elderly in Southwest China from 2013 to 2015. Medicine.

[46] Jeganathan S, Purnomo J, Houtzager L, Batterham M, Begley K (2010). Development and validation of a three-item questionnaire for dietitians to screen for poor oral health in people living with human immunodeficiency virus and facilitate dental referral. Nutr Diet.

[47] Khalifa N, Rahman B, Gaintantzopoulou MD, Al-Amad S, Awad MM (2020). Oral health status and oral health-related quality of life among patients with type 2 diabetes mellitus in the United Arab Emirates: a matched case-control study. Health Qual Life Outcomes.

[48] Nikbin A, Bayani M, Jenabian N, Khafri S, Motallebnejad M (2014). Oral health-related quality of life in diabetic patients: comparison of the Persian version of Geriatric Oral Health Assessment Index and Oral Health Impact Profile: a descriptive-analytic study. J Diabetes Metab Disord.

[49] Locker D, Matear D, Stephens M, Lawrence H, Payne B (2001). Comparison of the GOHAI and OHIP-14 as measures of the oral health-related quality of life of the elderly. Community Dent Oral Epidemiol.

[50] Masood M, Younis LT, Masood Y, Bakri NN, Christian B (2019). Relationship of periodontal disease and domains of oral health-related quality of life. J Clin Periodontol.

[51] Thomson WM, Williams SM, Broadbent JM, Poulton R, Locker D (2010). Long-term dental visiting patterns and adult oral health. J Dent Res.

[52] Leite FRM, Nascimento GG, Baake S, Pedersen LD, Scheutz F, López R (2019). Impact of smoking cessation on periodontitis: a systematic review and meta-analysis of prospective longitudinal observational and interventional studies. Nicotine Tob Res.

[53] Trevethan R (2017). Sensitivity, specificity, and predictive values: foundations, pliabilities, and pitfalls in research and practice. Front Public Health.

[54] Borgnakke WS (2019). IDF diabetes atlas: diabetes and oral health – a two-way relationship of clinical importance. Diabetes Res Clin Pract.

[55] Healthdirect: Cost of dental care. Australia: Healthdirect. 2021. https://www.healthdirect.gov.au/cost-of-dental-care. Accessed 25 May 2022.

[56] Leeflang MMG, Rutjes AWS, Reitsma JB, Hooft L, Bossuyt PMM (2013). Variation of a test's sensitivity and specificity with disease prevalence. CMAJ.

[57] Rajhans NS, Kohad RM, Chaudhari VG, Mhaske NH (2011). A clinical study of the relationship between diabetes mellitus and periodontal disease. J Indian Soc Periodontol.

[58] Zhang Y, Leveille SG, Shi L, Camhi SM (2021). Disparities in preventive oral health care and periodontal health among adults with diabetes. Prev Chronic Dis.

[59] The Royal Australian College of General Practitioners. Management of type 2 diabetes: a handbook for general practice. East Melbourne, Victoria: RACGP. 2020. https://www.racgp.org.au/getattachment/41fee8dc-7f97-4f87-9d90-b7af337af778/Management-of-type-2-diabetes-A-handbook-for-general-practice.aspx. Accessed 16 Feb 2022.

[60] Vergnes JN, Canceill T, Vinel A, Laurencin-Dalicieux S, Maupas-Schwalm F, Blasco-Baqué V, Hanaire H, Arrivé E, Rigalleau V, Nabet C, Sixou M, Gourdy P, Monsarrat P (2018). The effects of periodontal treatment on diabetic patients: the DIAPERIO randomized controlled trial. J Clin Periodontol.

[61] Girish Babu KL, Doddamani GM (2012). Dental home: patient centered dentistry. J Int Soc Prev Community Dent.

[62] Poudel P (2021). Diabetes and Oral Health (DIOH): a mixed-methods study to inform oral health care for people living with diabetes (the DIOH study) [dissertation on the Internet for a PhD].

